# Extracorporeal Cardiopulmonary Resuscitation for Management of Out-of-Hospital Cardiac Arrest in a Patient with Fulminant Myocarditis

**DOI:** 10.1155/2020/8881042

**Published:** 2020-08-07

**Authors:** Alexander J. Meyer, Michael A. Biersmith, Ernest L. Mazzaferri, Konstantinos Dean Boudoulas

**Affiliations:** Division of Cardiovascular Medicine, The Ohio State University, Columbus, Ohio, USA

## Abstract

A 68-year-old male with a witnessed out-of-hospital cardiac arrest while jogging who was managed with extracorporeal cardiopulmonary resuscitation (ECPR) is presented. The patient was found to be in refractory ventricular fibrillation by emergency medical service personnel and underwent advanced cardiac life support (ACLS) protocol with placement of an automated chest compression device. He was emergently transported to the cardiac catheterization laboratory. Due to refractory ventricular fibrillation, he was placed on venoarterial extracorporeal membranous oxygenation (VA-ECMO). Coronary angiography at that time showed nonobstructive coronary artery disease. Management with VA-ECMO and other supportive measures were continued for 5 days, after which a cardiac magnetic resonance imaging was performed with findings consistent with acute myocarditis. His condition substantially improved, and he was discharged from the hospital with good neurologic and functional status. Fulminant myocarditis is often fatal, but aggressive supportive measures with novel ECPR protocols may result in recovery, as it happened in this case.

## 1. Introduction

Myocarditis, an inflammation of the myocardial tissue, is not uncommon. Over two million cases are reported annually worldwide resulting in approximately 350,000 deaths [1, 2]. The pathophysiology, epidemiology, and disease course in myocarditis has been well described in the literature [3, 4]. Initial management is largely supportive, while long-term management consists of standard guideline-directed medical therapy for ventricular dysfunction, if present [3, 4]. Fulminant myocarditis that results in out-of-hospital cardiac arrest continues to have exceedingly high mortality [5]. Extracorporeal cardiopulmonary resuscitation (ECPR) at presentation represents a novel support strategy that has the potential to improve survival. A patient with refractory cardiac arrest due to fulminant myocarditis who was supported by ECPR and had excellent functional and neurologic recovery is presented.

## 2. Patient's Presentation

**Figure 1:**
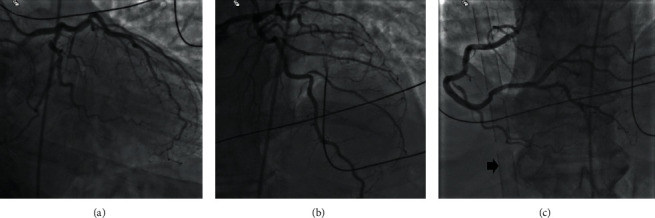
Coronary angiography on presentation. (a) Right anterior oblique and caudal projection and (b) right anterior oblique and cranial projection of the left coronary arteries demonstrating mild coronary artery disease. (c) Cranial projection of the right coronary artery demonstrating mild coronary artery disease; extracorporeal membranous oxygenation venous cannula originating from the femoral vein and extending into the right atrium can be seen (arrow).

A 68-year-old man had a witnessed cardiac arrest while jogging. Bystander cardiopulmonary resuscitation (CPR) was immediately initiated followed by advanced cardiac life support (ACLS) upon arrival of emergency medical service (EMS) personnel. The patient was noted to be in persistent ventricular fibrillation despite multiple defibrillations. A LUCAS chest compression system (Stryker Medical, Portage, Michigan) was applied and the patient was transported directly to the Ross Heart Hospital at The Ohio State University for ECPR. Upon arrival to the cardiac catheterization laboratory, the patient was still in ventricular fibrillation. He was placed on venoarterial extracorporeal membrane oxygenation (VA-ECMO) via the femoral artery and vein, as an adjunct to CPR, per our institution's ECPR protocol. A coronary angiography was then performed that demonstrated mild coronary artery disease (Figure 1). After initiation of VA-ECMO and administration of amiodarone and lidocaine infusions, ventricular fibrillation was converted to sinus rhythm, and the patient was transferred to the cardiac intensive care unit in critical condition. Supportive care including vasopressors and VA-ECMO were continued for 5 days. Hypothermia (35-36°C) was performed for 24 hours.

**Figure 2:**
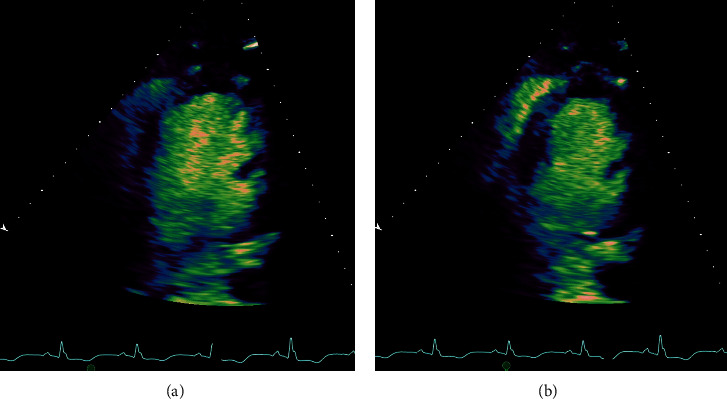
Transthoracic echocardiogram on admission demonstrating severe left ventricular (LV) systolic dysfunction with an ejection fraction of 20%. (a) Two-chamber view of LV end diastole and (b) LV end systole.

Initial electrocardiogram (ECG) showed sinus tachycardia with left intraventricular conduction defect. Laboratory testing results were notable for an initial lactate of 7.8 mmol/L and a peak troponin of 40 ng/mL. Transthoracic echocardiogram demonstrated severe left ventricle (LV) systolic dysfunction with an ejection fraction of 20% and a dilated LV (LV end diastolic and systolic volume indexes were 92 mL/m^2^ and 72 mL/m^2^, respectively) (Figure 2). Vasopressor support was eventually weaned off and on hospital day 5 VA-ECMO was discontinued and the patient was extubated. On hospital day 6, therapy with carvedilol was initiated. Angiotensin-converting enzyme inhibitor was not given due to renal dysfunction with a creatinine of 2.0 mg/dL. On hospital day 8, physical therapy was started at the bedside, and on the following day, the patient was ambulating in the hospital halls with the assistance of cardiac rehabilitation.

**Figure 3:**
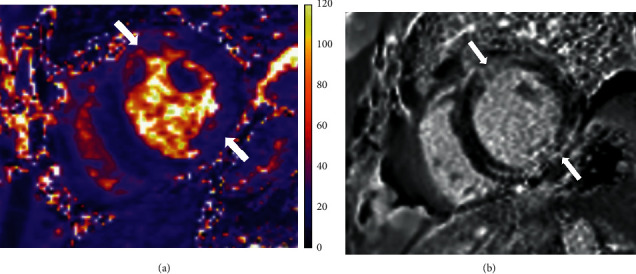
Cardiac magnetic resonance imaging of parasternal short axis. (a) T2-weighted images demonstrating extensive myocardial T2 elevation (arrow) of the left ventricle (LV) consistent with myocardial edema. (b) Extensive LV patchy, epicardial pattern of injury by late gadolinium enhancement (arrow) consistent with cellular membrane injury; there was no infarct scar seen. There was severe LV systolic dysfunction with an ejection fraction of 25%. These findings are consistent with a diagnosis of acute myocarditis.

Cardiac magnetic resonance imaging was obtained on hospital day 10 and demonstrated severe LV systolic dysfunction with an ejection fraction of 25%; there was also extensive epicardial late gadolinium enhancement and elevated T2 signal consistent with acute myocarditis (Figure 3). Prior to discharge, an implantable cardioverter defibrillator was placed on hospital day 11. The patient was discharged to home on hospital day 12 (home health care was provided). At that time, his cerebral perfusion category was 1 as he had full neurologic recovery. Discharge medications included carvedilol 12.5 mg twice daily, amiodarone 200 mg daily, furosemide 40 mg daily, aspirin 81 mg daily, and atorvastatin 20 mg daily.

At 30-day follow-up in heart failure clinic, the patient was doing well. He had no recollection of events immediately prior to his hospitalization or several days into the hospital course. He had no signs or symptoms of heart failure. The patient was walking one mile daily without symptoms. ECG findings demonstrated sinus rhythm with persistent left intraventricular conduction defect. Renal function that was impaired during the hospitalization was normal at this time, and therapy with losartan 25 mg twice daily was started. In addition, the medications that were initiated at hospital discharge were continued without dose adjustment. At 60 days after the patient's hospitalization, a repeat transthoracic echocardiogram was obtained that demonstrated a LV ejection fraction of 30% and LV end diastolic and systolic volume indexes of 77 mL/m^2^ and 32 mL/m^2^, respectively; these findings were improved compared to the initial echocardiogram.

## 3. Discussion

VA-ECMO is an extracorporeal (i.e., outside of body) technique that provides both cardiac and respiratory support sustaining life [6]. Using VA-ECMO as an adjunct to CPR, blood flow can be restored in patients with sustained cardiac arrest providing sufficient perfusion, importantly cerebral perfusion, to prevent fatal anoxic brain injury. The development of ECPR programs incorporating VA-ECMO as an adjunct to CPR has shown encouraging results in patients with out-of-hospital cardiac arrest demonstrating survival to hospital discharge ranging from 4% to 45% with good neurologic recovery (cerebral performance category score 1 or 2), though studies are limited [7–10].

In a study by Pozzi et al., 68 patients (43 ± 11 years) that underwent ECPR for refractory out-of-hospital cardiac arrest were included [11]. Patients were divided into two groups, shockable and nonshockable rhythms. The shockable rhythm group had a survival to hospital discharge of 31%, while there were no survivors in the nonshockable rhythm group [10]. Based on this and other previous experiences [10, 12, 13], it was determined by our group to focus our ECPR protocol on cardiac arrest due to refractory VT and/or VF, as was the case in the patient presented here.

A crucial component to an ECPR program is providing effective chest compressions in order to adequately perfuse the vital organs. Automated CPR devices have shown to improve cerebral blood flow compared to manual CPR and are critical to have in the field; automated CPR devices also play an important role during ambulance transport maintaining adequate compressions and for the safety of EMS personnel [14, 15].

Out-of-hospital cardiac arrest has exceedingly high mortality in all patients, as well as in patients with fulminant myocarditis. Long-term mortality outcomes in fulminant myocarditis are usually good if patients survive the initial hospitalization [16]. As most of the mortality due to fulminant myocarditis occurs during the acute phase of the disease, high levels of hemodynamic support at that time including an ECPR protocol are required [7, 8, 17]. Prior case series of VA-ECMO in cardiogenic shock caused by myocarditis have shown promising results demonstrating survival to hospital discharge ranging from 54% to 77% [11, 18]. However, in a case series of seven patients by Sieweke et al., refractory cardiogenic shock secondary to myocarditis caused by influenza was associated with poor survival despite use of VA-ECMO plus the percutaneous LV ventricular assist device Impella (ABIOMED, Danvers, Massachusetts), which the authors attributed to the multiorgan dysfunction often seen with severe influenza infection [19].

Implementation of an ECPR program in patients with fulminant myocarditis and cardiac arrest can prove to be life-saving, as was the case with the patient presented here. Due to limited experience on the topic, a national or international registry would be useful to further define the role of ECPR in the care of these patients.
